# Biomarkers for Hepatocellular Carcinoma

**DOI:** 10.1155/2012/859076

**Published:** 2012-05-10

**Authors:** Tara Behne, M. Sitki Copur

**Affiliations:** ^1^College of Pharmacy, University of Nebraska Medical Center, Omaha, NE 68198, USA; ^2^Saint Francis Cancer Treatment Center, Grand Island, NE 68803, USA

## Abstract

The hepatocellular carcinoma (HCC) is one of the most common malignant tumors and carries a poor survival rate. The management of patients at risk for developing HCC remains challenging. Increased understanding of cancer biology and technological advances have enabled identification of a multitude of pathological, genetic, and molecular events that drive hepatocarcinogenesis leading to discovery of numerous potential biomarkers in this disease. They are currently being aggressively evaluated to establish their value in early diagnosis, optimization of therapy, reducing the emergence of new tumors, and preventing the recurrence after surgical resection or liver transplantation. These markers not only help in prediction of prognosis or recurrence but may also assist in deciding appropriate modality of therapy and may represent novel potential targets for therapeutic interventions. In this paper, a summary of most relevant available data from published papers reporting various tissue and serum biomarkers involved in hepatocellular carcinoma was presented.

## 1. Introduction

As molecular indicators of biological status, biomarkers, detectable in blood, urine, or tissue, can be useful for the clinical management of various disease states. Threshold concentrations can be utilized to identify the presence of various diseases. Concentration fluctuations have the potential to guide therapy in disease progression. Numerous biomarkers have been identified for various disease states. Research is ongoing to fully understand and evaluate the clinical significance of utilizing biomarkers. Time and money can be saved by avoiding empiric or broad treatment approaches to diseases of particular organs or systems, and ideally, biomarkers could serve as a measurement tool to detect disease presence and progression and to guide more targeted therapy. Many disease states, especially various types of cancer, can be better understood by the utilization of tumor biomarkers. Hepatocellular carcinoma (HCC) is one such cancer that can benefit from tumor biomarkers' diagnostic, therapeutic, and prognostic capabilities.

HCC is the fifth most common malignant tumor and the third leading cause of cancer-related deaths. Worldwide, there are about 626,000 new HCC cases and nearly 600,000 HCC-related deaths each year with an incidence equal to the death rate [1, 2]. Although the molecular mechanisms by which HCC develops remain largely unclear, a multitude of pathological, genetic, and molecular events that drive hepatocellular carcinogenesis has been identified.

Current gold standard and most commonly used biomarkers for patients at risk for HCC, alpha-fetoprotein (AFP) along with ultrasound every 6 to 12 months, is far from perfect. Serum AFP levels of more than 400 ng/mL are considered diagnostic; however, such high values are observed only in a small percentage of patients with HCC. Ultrasound surveillance even performed at every three monthly intervals cannot improve detection of small HCC because of limitations in recall procedures [3, 4].

With advances in understanding of tumor biology, along with the development of cellular and molecular techniques, the role of biomarkers related to early detection, invasiveness, metastasis, and recurrence has attracted great deal of research interest resulting in discovery and utilization of several novel markers in this disease. In this paper we try to give an overview of available data on this burgeoning area of research.

## 2. Biomarkers for Liver Cancer

### 2.1. Oncofetal and Glycoprotein Antigens

#### 2.1.1. Alpha-Fetoprotein (AFP)

The first serologic assay for detection and clinical followup of patients with hepatocellular carcinoma was alpha-fetoprotein (AFP) which has been the standard tumor biomarker for HCC for many years. It is a glycoprotein produced by the fetal liver and yolk sac during pregnancy. Serum AFP levels are often elevated in HCC, but this is not always the case. AFP levels may be elevated initially in the early stages of HCC and then drop or even normalize before rising again as disease progression occurs [5]. Additionally, AFP elevation has also been recognized in the presence of acute and chronic viral hepatitis as well as in patients with cirrhosis caused by hepatitis C. Given the multiple indications that present with elevated AFP levels, it is necessary to evaluate the significance of serum concentrations. In general, consistently elevated serum AFP levels greater than 500 ng/mL are indicative of HCC. Lower serum concentrations which are only transient in nature are more often present in benign liver disease [6]. If a patient has known risk factors for HCC, such as the presence of cirrhosis, increasing levels of AFP have been shown to correlate with the development of HCC [6]. Unfortunately, AFP serum concentrations do not correlate well with the prognostic values of HCC such as tumor size, stage, or disease progression, and ethnic variability may also exist. Furthermore, in some cases of HCC, AFP elevations are not apparent at all [7]. Total AFP can be divided into three different glycoforms, AFP-L1, AFP-L2, and AFP-L3-based on their binding capability to lectin *Lens culinaris* agglutinin (LCA). High percentage of AFP-L3 has been shown to be associated with poor differentiation and biologically malignant characteristics, worse liver function, and larger tumor mass [8].

#### 2.1.2. Glypican-3

Glypican-3 (GPC3), a membrane-anchored heparin sulfate proteoglycan, has been demonstrated to interact with growth factors and modulate their activities. It binds to the cell membrane through the glycosylphosphatidylinositol anchors. GPC3 mRNA was upregulated significantly in tumor tissues of HCC compared to paraneoplastic liver tissue, liver tissues of healthy adults, and liver tissues of patients with nonmalignant hepatopathy. The expression of GPC3 (at both mRNA and protein levels) in the serum of HCC patients was significantly higher than that in the serum of healthy adults or patients with nonmalignant disease. It can be detected in 40–53% of HCC patients and 33% of HCC patients seronegative for both AFP and Des-gamma-carboxyprothrombin (DCP) [9, 10]. It has been shown that soluble GPC3 (sGPC3), the NH_2_-terminal portion of GPC3, is superior to AFP in the sensitivity of detecting well or moderately differentiated HCC, and the simultaneous determination of both markers improves overall sensitivity from 50% to 72%. Recently, a study compared the survival rate between the GPC3-positive and GPC3-negative HCC patients. GPC3 positivity correlated with poor prognosis and identified as an independent prognostic factor for the overall survival on multivariate analysis [11].

### 2.2. Enzymes and Isoenzymes

#### 2.2.1. Des-Gamma-Carboxy (Abnormal) Prothrombin (DCP)

DCP is produced by the malignant hepatocyte and appears to result from an acquired posttranslational defect in the vitamin-K-dependent carboxylase system. DCP production is independent of vitamin K deficiency, although pharmacological doses of vitamin K can transiently suppress DCP production in some tumors. DCP levels greater than 0.1 AU/mL (100 ng/mL) on ELISA are highly suggestive of HCC or tumor recurrence. Normalization of DCP levels correlates well with successful tumor resection and appears to be an excellent marker of tumor activity. It is thought that the combination of AFP and DCP assays will increase the sensitivity of testing. The correlation between tumor size and DCP levels is not yet clearly defined. It appears that there is a correlation in DCP levels and large tumors; however, the same is not the case in small tumors (<3 cm) [15]. A cross-sectional case control study involving 207 patients determined that DCP is more sensitive and specific than AFP for differentiating HCC from nonmalignant liver disease. In this study there were 4 groups studied: normal healthy subjects; patients with noncirrhotic chronic hepatitis, patients with compensated cirrhosis, and patients with histologically proven HCC. Both DCP and AFP levels increased among the groups as disease severity increased (from normal to HCC), but DCP values had less overlap among the groups than AFP. Study results concluded that a DCP value of 125 mAU/mL yielded the best sensitivity and specificity for differentiating patients with HCC from those with cirrhosis and chronic hepatitis [16]. Sensitivity and specifity of total AFP, AFP glycoforms, DCP, and combinations of both markers have been summarized in Table 1.

#### 2.2.2. Gamma-Glutamyl Transferase

Serum gamma-glutamyl transferase (GGT) in healthy adults is mainly secreted by hepatic Kupffer cell and endothelial cell of bile duct, and its activity increases in HCC tissues. Total GGT can be divided into 13 isoenzymes by using polymer acrylamide gradient gel electrophoresis, and some of them can only be detected in the serum of HCC patients. Sensitivities of GGTII have been reported to be 74.0% in detecting large HCC and 43.8% in detecting small HCC. Sensitivity can be significantly improved with the simultaneous determination of GGTII, DCP, and AFP [17].

#### 2.2.3. Serum Alpha-1-Fucosidase

 Alpha-l-fucosidase (AFU) is a lysosomal enzyme found in all mammalian cells with a function to hydrolyze fucose glycosidic linkages of glycoprotein and glycolipids. Its activity increases in the serum of HCC patients (1418.62 ± 575.76 nmol/mL/h) compared with that in the serum of healthy adults (504.18 ± 121.88 nmol/mL/h, *P* < 0.05), patients with cirrhosis (831.25 ± 261.13 nmol/mL/h), and patients with chronic hepatitis (717.71 ± 205.86 nmol/mL/h). It has been reported that the sensitivity and specificity of AFU at the cut-off value of 870 nmol/mL/h were 81.7% and 70.7%, respectively [18]. AFU measurement is useful in association with AFP in early diagnosis of HCC and could serve as a valuable supplementary to AFP. It has been indicated that HCC will develop within few years in 82% of patients with liver cirrhosis, if their serum AFU activity exceeds 700 nmol/mL/h. The activity of AFU was reported to be elevated in 85% of patients at least 6 months before the detection of HCC by ultrasonography [19].

#### 2.2.4. Human Carbonyl Reductase 2

This enzyme expressed in the human liver and kidney is important in detoxification of the reactive alpha-dicarbonyl compounds and reactive oxygen species deriving from oxidative stress in HCC. The human carbonyl reductase 2 levels have been shown to be inversely correlated to the pathological grading of HCC [20].

#### 2.2.5. Golgi Phosphoprotein 2

Golgi phosphoprotein 2 (GOLPH2), a Golgi-apparatus-associated protein, has been shown to have a higher sensitivity than AFP in the detection of HCC [21]. A recent study found that GOLPH2 protein was highly expressed in tissues of HCC (71%) and bile duct carcinoma (85%) patients. GOLPH2 protein levels were detectable and quantifiable in sera by ELISA. In patients with hepatitis C, serial ELISA measurements in the course of the disease appear to be a promising complimentary serum marker in the surveillance of HCC [22].

### 2.3. Growth Factors and Their Receptors

#### 2.3.1. Transforming Growth Factor-Beta (TGF-Beta)

Belonging to a superfamily of polypeptide signaling molecules involved in regulating cell growth, differentiation, angiogenesis, invasion, and immune function, TGF-beta is a predominant form of growth factor family in humans. Its mRNA and protein are overexpressed in HCC compared with surrounding liver tissues, especially in small and well-differentiated HCCs [23]. However, no relationship has been shown between TGF-beta expression and posthepatectomy survival [24]. Serum TGF-beta level has been found to be elevated in HCC patients compared to healthy adults or patients with nonmalignant liver disease [25–27].

#### 2.3.2. Tumor-Specific Growth Factor (TSGF)

Malignant tumors release tumor-specific growth factor (TSGF) into peripheral blood during their growing period. Serum levels of TSGF may reflect the existence of tumor. TSGF can be used as a diagnostic marker in detecting HCC, and its sensitivity can reach 82% at the cut-off value of 62 U/mL and may have a higher accuracy with the simultaneous determination of other tumor markers. The simultaneous determination of TSGF (at the cut-off value of 65 U/mL), AFP (at the cut-off value of 25 ng/mL), and serum ferritin (at the cut-off value of 240 ng/mL) can reach a sensitivity and specificity of 98.4% and 99%, respectively [26].

#### 2.3.3. Epidermal Growth Factor Receptor Family

The epidermal growth factor receptor (EGFR) family consists of four closely related transmembrane tyrosine kinase receptors: EGFR (erbB-1), c-erb-2 (Her-2/neu), c-erb-3 (HER-3), and c-erb-4 (HER-4). These bind with ligands of the EGF family, including EGF, TGF-alpha, and heparin-binding EGF. High levels of EGFR expression have been associated with early recurrence and reduced disease-free survival following resection of hepatocellular carcinoma [27].

#### 2.3.4. Hepatocyte Growth Factor/Scatter Factor

Hepatocyte growth factor/scatter factor (HGF/SF) is a cytokine with a wide range of effects from embryonic development and liver regeneration. It is associated with molecular mechanisms of hepatocarcinogenesis via paracrine system involving its cellular receptor, c-met. High c-met expression has been shown in invasive-type HCC and has been associated with metastasis and reduced overall survival [28, 29].

#### 2.3.5. Basic Fibroblast Growth Factor

 This is a soluble heparin-binding polypeptide with a potent mitogenic effect on endothelial cells. Elevated levels above the median of >10.8 pg/mL have been shown to predict decreased disease-free survival [30]. Recent preliminary data with targeted therapy lenalidomide which inhibits fibroblast growth factor (FGF) showed promising and in some patients dramatic activity in HCC patients [31].

### 2.4. Molecular Markers

#### 2.4.1. Circulating Nucleic Acids: mRNAs

The analysis of circulating nucleic acids in plasma offers another avenue for noninvasive monitoring of a variety of physiological and pathologic conditions [30, 31]. Numerous applications based on the detection of circulating cell-free nucleic acids in human plasma have been reported for the management of malignancies. The fundamental principle underlying these applications relates to the detection in plasma of extracellular nucleic acid molecules derived from diseased organs. Analysis of cell-free plasma RNA offers an opportunity for the development of pathology-related markers [32–34].

**Alpha-Fetoprotein mRNA (AFP mRNA)**

Matsumura et al. first reported that single HCC cell could be detected in circulation by means of reverse-transcription polymerase chain reaction (RT-PCR), targeting AFP mRNA [35]. This led to further reports of the value of AFP mRNA as a predictor for HCC recurrence. Rather controversial results were attributed to the blood borne dispersion of both tumor cells and normal liver cells and the mistranscription of mRNA encoding AFP by peripheral mononuclear cells. The recurrence-free interval of HCC patients with postoperative serum AFP mRNA positivity has been reported to be significantly shorter than that of HCC patients with postoperative negativity (53% versus 88% at 1 year, 37% versus 60% at 2 years, *P* = 0.014) [34] and (52.6% versus 81.8% at 1 year, 15.6% versus 54.5% at 2 years, and 0% versus 29.2% at 3 years, *P* < 0.001) [36]. A meta-analysis showed that the expression of AFP mRNA one week after surgery was correlated with the recurrence of HCC [37].

**Gamma-Glutamyl Transferase mRNA (GGT mRNA)**

Similar to AFP, GGT mRNA can be detected in the serum and liver tissues of healthy adults, patients with liver disease, benign liver tumor, HCC, and secondary tumors of the liver [38]. The two types of GGT mRNA, type A and type B, have been identified. Type B is the predominant one in cancerous tissue suggesting that changes in the expression of hepatic GGT mRNA may be related to the development of HCC [39]. Patients with HCC harboring type B GGT mRNA both in cancer and in noncancerous tissue had a worse outcome, earlier recurrence, and more recurrence-related mortality. The presence of type B GGT mRNA in cancerous tissue was statistically correlated with high serum level of AFP, daughter nodules, higher postresection recurrence rate than those without it (63.6% versus 14.3%), and lower postrecurrence survival. The presence of type B GGT mRNA in non-cancerous liver tissue was significantly correlated with hepatitis C infection, high serum level of AFP, absence of infiltration of capsule, vascular permeation, daughter nodules, postresection recurrence, and postrecurrence survival [40].

**Insulin-Like Growth Factor II (IGF-II) mRNA**

Abnormal expression of IGF-II mRNA can be a useful tumor marker for diagnosis, differentiation, extrahepatic metastasis, and monitoring of postoperative recurrence in HCC. The determination of serum insulin-like growth factor-II (IGF-II) (at the cut-off value of 4.1 mg/g, prealbumin) has a sensitivity of 63%, specificity of 90%, and accuracy of 70% in the diagnosis of small HCC [41]. It can be a complementary tumor marker to AFP for diagnosis of small HCC. The simultaneous determination of IGF-II and AFP (at the cut-off value of 50 ng/mL) can improve the sensitivity to 80% and accuracy to 88% [42].

**Albumin mRNA**

Albumin is the most abundant protein in the body synthesized by the liver. mRNA of albumin is detectable in human plasma and could be a diagnostically sensitive marker for liver pathologies. Extracellular-based assays (circulating DNA/RNA) have been found to be better than cell-based assays (circulating tumor cells) in detection of preneoplastic lesions and micrometastases as plasma levels of circulating cancer-derived nucleic acid are higher than the levels of circulating cancer cells and are less prone to sampling errors. Cheung and colleagues studied the preoperative plasma samples obtained from 72 HCC patients who had undergone liver transplantation and found that patients with plasma albumin mRNA level (>14.6) had a significantly higher recurrence rate on multivariate analysis. High plasma albumin mRNA level predicted the 2-year recurrence rate with sensitivity and specificity of 73% and 70%, respectively [43].

**MicroRNAs (miRNAs)**

MicroRNAs (miRNAs) are a family of endogenous, small (21–23 nucleotides), noncoding but functional RNAs, which have been found in worms, flies, and mammals including human beings [44]. It is estimated that there are about 1,000 miRNA genes in the human genome with approximately 500 miRNA genes being already identified [45]. Similar to mRNA, HCC-associated miRNAs could be used as diagnostic and prognostic biomarkers of HCC with a potential for even greater accuracy. MiRNAs can accurately predict whether liver cancer will spread and whether liver cancer patients will have shorter or longer survival. MicroRNAs regulate gene expression by binding to specific messenger RNAs and prevent their translation into protein. Because each type of miRNA is able to downregulate hundreds of genes at a time, they can control entire transcriptional programs that determine fundamental cellular properties and behavior. Accordingly, miRNA profiling has emerged as an extremely valuable method for phenotyping and subclassifying tumors [44]. Compared to conventional gene expression profiling (in which protein-coding, messenger RNAs are examined), miRNA analysis has several advantages. Due to the stability of miRNAs, formalin-fixed samples (rather than frozen tissue) can be used. Furthermore, the interrogation of hundreds of miRNAs (and often significantly fewer) yields as much information as might be gleaned from examining thousands of messenger RNAs.

Many independent groups have conducted comprehensive analyses of miRNAs in HCC, and a plethora of information on miRNA markers has been identified. Many of these miRNA signatures correlate with important biological parameters, such as metastasis [46–48], differentiation [49–51], HBV or HCV infection [52, 53], tumor recurrence [54], and patient survival [55, 56]. Some miRNAs are involved in HCC carcinogenesis by promoting cancer stem cell and by controlling cell proliferation and apoptosis; others are associated with HCC progression by controlling cell migration and invasion. These HCC-associated miRNAs not only provide new insights into the molecular basis of HCC but also serve as new tools for HCC diagnosis and prognosis. Currently a few miRNA signatures, however, could potentially be used in this area. Some miRNAs have been validated in an independent cohort, paving the way for clinically useful platforms to assess HCC risk and outcome. This promising area of research awaits further validation in prospective studies [57].

### 2.5. Pathological Biomarkers

Finally there have been reports of pathological biomarkers of HCC for diagnosis and prognosis. Some of these diagnostic biomarkers focus on immunochemical staining patterns to distinguish high-grade dysplastic nodules and well-differentiated HCC. The best type of immunostaining for this difficult condition has been reported to be the combination of heat-shock protein 70 (HSP70), glypican-3 (GPC3), and glutamine synthetase (GS). For prognostic use a number of histological and immuno-histochemical markers such as markers of cell proliferation (Ki67), apoptosis or cell survival (survivin), cell adhesion molecules (E-cadherin), neoangiogeneis (VEGF), and more have been looked in small studies showing promise; however, most of these markers have not been validated in large studies [57]. Various HCC biomarkers and their clinical use have been summarized in Table 2.

## 3. Discussion

Hepatocarcinogenesis is a complex multistate process usually occurring after many years of chronic exposure to several mitogenic and mutagenic environments precipitating random genetic alterations. Recent evidence suggest that intrinsic biologic characteristics of the tumor in terms of proliferation and invasiveness are probably related to different composition and activity of the microenvironment, leading to very different clinical outcomes. HCC is rather unique with its ability to synthesize various tumor-related proteins rendering itself more suitable to biomarker-related research than other tumors. Because of the large multitude of biomarkers reported in this disease, selecting the biomarkers which would be most useful in clinical practice has been more than challenging. In this rather brief overview, we tried to focus on most widely used and accepted biomarkers.

Despite its limitations, serum AFP still remains the most widely used tumor marker in clinical practice. Recent research favors the circulating hepatoma-specific AFP subfraction AFP-L3 and DCP over AFP alone in differentiating HCC from nonmalignant hepatopathy and detecting small HCC. Furthermore, some other tumor markers, such as GPC3, GGT II, AFU, have been shown to be supplementary to AFP and DCP in the detection of HCC. Some of them even can be detected in HCC patients seronegative for both AFP and DCP, thus indicating that the simultaneous determination of these markers may improve the accuracy.

However, most exciting and promising area of research in this disease has been the identification of a new group of molecules called miRNAs. MiRNAs have been discovered to be aberrantly expressed in HCC, and some of them are functionally involved in HCC carcinogenesis and progression. Furthermore, certain microRNAs are associated with HCC or related to HCC subtypes, implicating the potential use of microRNAs in HCC patient stratification of diagnosis and prognosis. Some of these HCC-associated miRNAs have been validated in independent cohorts. This brings the possibility of developing clinically useful platforms to develop HCC diagnosis, risk assessment, and patient risk stratification with the ultimate goal of personalized therapy.

## 4. Conclusion

 Research into the molecular biology of hepatocarcinogenesis has identified numerous biomarkers which could provide additional information for HCC biologic behavior metastasis and recurrence to that gained from traditional histopathological features. A large number of biomarkers have been shown to have potential predictive significance. However, most of them have been studied retrospectively. Efforts should be directed towards prospective clinical trials in evaluating the prognostic significance of these markers. These molecules not only help in prediction of prognosis for patients with HCC but may also assist in deciding appropriate modality of therapy and represent novel targets for therapeutic interventions.

## Figures and Tables

**Table 1:** Diagnostic values of HCC serum markers [12–14].

Type of test	Sensitivity (%)	Specificity (%)
AFP-L3	61.6	92.0
DCP	72.7	90.0
AFP	67.7	71.0
AFP-L3 + DCP	84.8	97.8
AFP-L3 + AFP	73.7	86.6
DCP + AFP	84.8	90.2
AFP-L3 + DCP + AFP	85.9	59.0

**Table 2:** Various HCC biomarkers and their clinical use.

HCC marker	Clinical use
Alpha-fetoprotein	Early diagnosis, monitoring, and recurrence
*Lens culinaris* agglutinin reactive AFP (AFP-L3%)	Early diagnosis and prognosis, vascular invasion
Des-gamma-carboxy prothrombin (DCP)	Early diagnosis and prognosis, portal vein invasion and metastasis
Gamma-glutamyl transferase	Early diagnosis complementary to other markers
Alpha-l-fucosidase	Early diagnosis
Glypican-3	Early diagnosis
Human carbonyl reductase 2	Prognosis
Golgi phosphoprotein 2	Tumor aggressiveness
Transforming growth factor beta	Tumor invasiveness
Hepatocyte growth factor (HGF)	Prognosis and disease recurrence
Transforming growth factor-b (TGF-b)	Prognosis invasiveness
Tumor specific growth factor	Diagnosis complementary to other markers
Epidermal growth factor receptor family	Early recurrence
Hepatocyte growth factor	Metastasis reduced survival
Micro RNAs	Tumor spread and survival
